# Diagnosis and treatment progress of upper eyelid abnormalities in thyroid-eye disease

**DOI:** 10.3389/fendo.2026.1860864

**Published:** 2026-07-13

**Authors:** Cheng Gai, Bing Wang

**Affiliations:** Department of Ophthalmology, Shandong Provincial Hospital Affiliated to Shandong First Medical University, Jinan, Shandong, China

**Keywords:** biological products, medical treatment, surgical treatment, thyroid eye disease, upper eyelid abnormalities

## Abstract

Thyroid eye disease (TED) is the most common orbital disorder among adults. Among its many clinical features, upper eyelid abnormalities—namely, retraction, lid lag, and swelling—frequently cause functional and cosmetic problems. This article reviews current knowledge on diagnosing and treating these abnormalities. We examine a range of options, from basic supportive care and local injections (botulinum toxin A, hyaluronic acid, and triamcinolone) to systemic drugs (corticosteroids, Teprotumumab, and alpha-1 antagonists) and surgery (levator recession, blepharotomy, and orbital decompression). Recent data suggest that IGF-1 receptor antagonists, especially Teprotumumab, can substantially reduce eyelid retraction and proptosis with a better safety profile than traditional glucocorticoids. Nevertheless, important questions remain: few head-to-head trials exist, the optimal timing for biologics versus surgery is unclear, and treatment needs to be individualized. Future studies should integrate artificial intelligence for quantitative eyelid assessment and promote multidisciplinary care. This review offers a practical framework for clinicians and outlines their research priorities.

## 1 Introduction

Thyroid eye disease (TED), also known as Graves’ ophthalmopathy (GO) or thyroid-associated ophthalmopathy (TAO), is an autoimmune disorder closely associated with thyroid dysfunction and the most prevalent orbital disease in adults (1). Its clinical manifestations are diverse, affecting multiple orbital structures including the eyelids, extraocular muscles, lacrimal glands, and orbital fat. Disease progression may lead to proptosis, eyelid abnormalities, diplopia, exposure keratitis, and compressive optic neuropathy, resulting in irreversible visual impairment or blindness. The etiology remains complex, with no consensus; studies suggest that TED development is primarily linked to environmental, genetic, and immunological factors (2). Among its various manifestations, upper eyelid abnormalities are one of the most common presentations, primarily eyelid retraction, lagging eyelid movement, and eyelid swelling, occurring in up to 70%-90% of cases. These abnormalities severely impair patients’ visual function, corneal integrity, and quality of life. In recent years, with advancing understanding of the pathogenesis of TED, the roles of the levator palpebrae superioris rectus complex (LPS-SR complex) and the “fixation duress” mechanism secondary to inferior rectus muscle restriction in the development of TED-related upper eyelid abnormalities have garnered significant attention (3).

## 2 Epidemiology and clinical significance of TED

Thyroid eye disease (TED) is the most prevalent orbital disorder in adults. According to the literature, approximately 25%-30% of patients with Graves’ hyperthyroidism develop Graves’ ophthalmopathy (4). The annual incidence rate is 0.54–0.9 cases per 100,000 men and 2.67–3.3 cases per 100,000 women (5). Eyelid abnormalities, as the most common clinical manifestation of TED, have a significant diagnostic value. In a case–cohort study, Bartley et al. (6) found that over 90% of patients with TED exhibited unilateral or bilateral eyelid retraction during the disease course, with upper eyelid retraction being particularly common. This clinical finding serves not only as a critical basis for TED diagnosis but also as an indicator for assessing disease severity and treatment efficacy. Upper eyelid abnormalities encompass various symptoms, including eyelid edema, delayed eyelid closure, and palpebral conjunctival hyperemia with upper eyelid retraction. Upper eyelid retraction is the most challenging symptom to correct; it is typically defined as an upper eyelid margin of more than 2 mm above the limbus in the primary gaze position (1). When the upper eyelid margin reaches or exceeds the upper limbus, it can lead to incomplete blinking and sleep with open eyes, subsequently causing exposure keratitis. In severe cases, it may progress to corneal ulceration or even corneal perforation. Additionally, the “staring” or “surprised” facial expression caused by upper eyelid retraction often induces social anxiety and psychological distress in patients, significantly impairing their quality of life (7).

## 3 Pathogenesis of upper eyelid abnormalities at TED

### 3.1 Müller’s muscle-related mechanism

Müller’s muscle is a smooth muscle innervated by the sympathetic nervous system (8) and serves as an auxiliary muscle that assists in eye opening by elevating and maintaining the upper eyelid. The traditional view holds that elevated thyroid hormone levels enhance sympathetic nervous system activity, leading to excessive contraction of Müller’s muscle and consequently causing eyelid retraction (9, 10). This mechanism explains why some patients experience aggravated eyelid retraction when the hyperthyroidism is poorly controlled. However, the sympathetic excitation theory alone cannot account for all clinical manifestations, such as the presence of unilateral retraction and persistent retraction during disease quiescence. Cockerham et al. (11) compared Müller’s muscle in patients with varying degrees of upper eyelid retraction (TED) with that in patients undergoing trauma or orbital exenteration. They observed significant fibrosis, mast cell infiltration, and numerous contracted Müller’s muscle cells under light microscopy in the TED group, with positive immunohistochemical staining for leukocyte common antigen, indicating inflammatory changes. Thus, they concluded that upper eyelid retraction in TED patients is associated with Müller’s muscle tissue fibrosis, fat infiltration, inflammatory responses, and focal atrophy of Müller’s muscle. Shih et al. (12) investigated Müller’s muscle in 31 patients with Müller’s muscle resection-induced TED and found that the degree of fibrosis was correlated with the severity of upper eyelid retraction. In fibrotic areas, there was increased macrophage infiltration and upregulated expression of macrophage colony-stimulating factor mRNA, and elevated levels of peroxisome proliferator-activated receptor-γ mRNA were associated with adipose infiltration. Reduced muscle mass correlated with decreased myocardial protmRNA expression; and decreased c-kit expression in the affected muscles coincided with reduced mast cell counts. This study suggests that the reduction in Müller’s muscle mass may result from impaired myogenesis, increased lipogenesis, and potential transdifferentiation of myocytes into adipocytes, indicating that Müller’s muscle may serve as a critical target in TED autoimmunity.

### 3.2 LPS-SR complex-related mechanisms

The LPS-SR complex served as the primary driving force for active upper eyelid elevation. During the active phase of TED, autoimmune inflammation of the orbital tissues may involve the levator palpebrae superioris muscle, leading to muscle fiber hypertrophy and hyperexcitability. In the stationary phase, post-inflammatory fibrotic changes cause adhesions between the levator palpebrae superioris and the superior rectus aponeurosis and surrounding tissues (such as orbital septum and orbicularis oculi muscle), restricting eyelid downward movement (13, 14). Additionally, fibrosis of the inferior rectus muscle constitutes a significant driver of levator palpebrae superioris hyperactivity (15, 16). According to Hering’s law, the bilateral synergistic muscles receive equal neural impulses. When fibrotic changes in the inferior rectus limit downward eye movement, the LPS-SR complex requires excessive neural excitation to overcome this limitation, which result in eyelid retraction and lag. Ohnishi et al. (17) measured LPS-SR complex thickness in patients with TED via MRI and found significantly thicker complexes compared with healthy controls.

### 3.3 Exophthalmos and mechanical factors

Since patients with TED often present with abnormal proliferation of orbital fat and hypertrophy of the extraocular muscles, leading to proptosis, severe proptosis can exacerbate upper eyelid retraction. When the eyeball protrudes forward, the upper eyelid covers the ocular surface. If the length of the tarsal plate and eyelid tissues is insufficient, it inevitably causes upward displacement of the eyelid margin (18), resulting in lagging of the upper eyelid and incomplete coverage of the ocular surface, which can lead to symptoms such as dry eye and corneal ulcers. This mechanism also explains why some patients exhibit significant improvement in upper eyelid retraction after orbital decompression surgery.

### 3.4 Thyroid-stimulating hormone receptor and insulin-like growth factor-1 receptor factors

Recent studies have suggested that orbital fibroblasts (OFs), as targets of TED immune attacks, express thyroid-stimulating hormone receptors (TSHR) and insulin-like growth factor-1 receptors (IGF-1R) on their surfaces, which play pivotal roles in the pathogenesis of upper eyelid abnormalities (1, 19, 20). Immunohistochemical studies have demonstrated the overexpression of TSHR in orbital tissues of patients with TED. TSHR activation induces the differentiation of orbital fibroblasts and pre-adipocytes into adipocytes, promoting proliferation of orbital adipose tissue. IGF-1R has also been shown to stimulate the proliferation of orbital fibroblasts in patients with TED and enhance the secretion of hyaluronic acid and cytokines. Notably, IGF-1R and TSHR form a signaling complex (21), which activates OFs, these activated OFs produce abundant inflammatory factors, differentiate into myofibroblasts, and secrete collagen, leading to fibrosis, contracture, and shortening of the extraocular and eyelid retractor muscles (superior levator palpebrae superioris and inferior rectus muscle), resulting in upper eyelid swelling and retraction.

**Figure 1:**
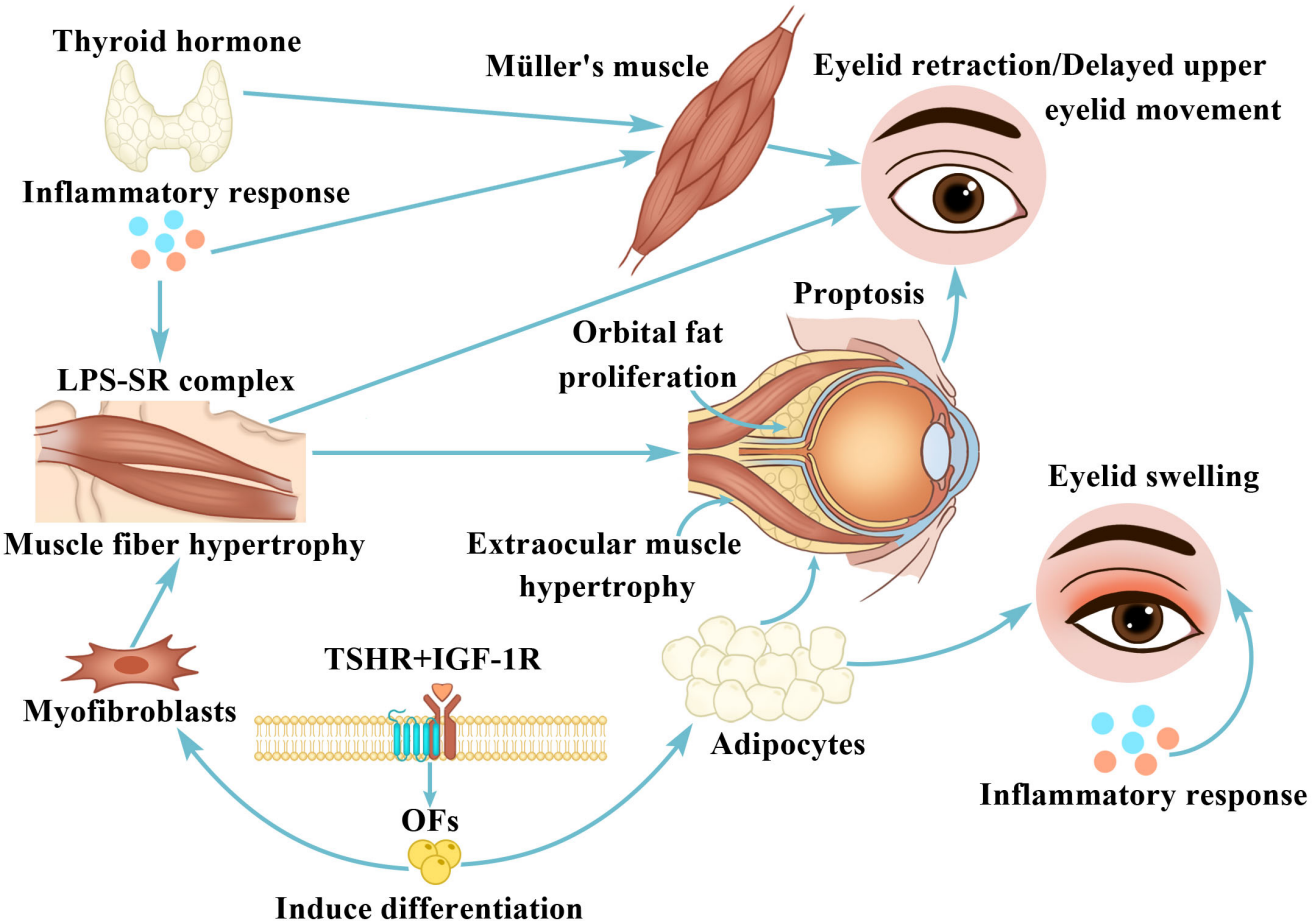
Schematic diagram of the pathogenesis of TED-related upper eyelid abnormalities.

**Figure 1** illustrates the main pathogenic mechanisms of upper eyelid abnormalities in TED (upper eyelid retraction, eyelid lag, and eyelid swelling). These mechanisms include excessive contraction or pathological changes of the Müller muscle induced by thyroid hormones and inflammatory factors, inflammation and fibrosis of the LPS-SR complex, mechanical effects caused by proptosis, and the interaction between IGF1R and TSHR on OFs.

## 4 Evaluation and measurement criteria for upper eyelid abnormalities

Accurate assessment and measurement are critical for formulating treatment plans and evaluating therapeutic efficacy. Current advancements in the assessment and measurement of upper eyelid abnormalities in patients with TED have primarily focused on quantitative imaging techniques. Traditional measurement methods include measuring the margin reflex distance 1 (MRD1)—distance from the upper eyelid margin to the corneal reflection point, and palpebral fissure height (PFH), which represents the maximum vertical distance between the upper and lower eyelid margins at the eye level. The application of clinical photogrammetric software enhances the measurement accuracy and reproducibility. However, the CAS score remains a pivotal method for evaluating the overall activity and severity of TED, particularly in determining whether upper eyelid abnormalities are in an inflammatory active phase, which directly influences treatment strategy selection. Infrared meibography can assess the severity of meibomian gland dysfunction in patients with TED, as incomplete blinking and ocular surface exposure caused by upper eyelid abnormalities exacerbate this condition (22). Anterior segment-optical coherence tomography (AS-OCT) enables high-resolution scanning of the eyelid cross section, improving the precision of measurements for levator palpebrae superioris muscle thickness, Müller’s muscle thickness, and their distances from the eyelid margin, thereby providing an objective anatomical basis for evaluating upper eyelid abnormalities. High-resolution MRI has also been employed to monitor ocular changes in patients (23). It not only clearly displays the morphology and volume of extraocular muscles but also enables quantitative assessment of muscle inflammatory edema and blood supply through T2-weighted sequence signal intensity and contrast-enhanced imaging, facilitating the differentiation between active inflammation and chronic fibrosis, as well as predicting responses to immunotherapy and postoperative outcomes (24). Some studies have demonstrated the significance of rs-EPI-based DTI in evaluating microstructural alterations of extraocular muscles and the optic nerve in TED, whereas quantitative MRI assessment of ocular muscle structures further aids in TED diagnosis and staging (25, 26). Recently, researchers have pioneered the application of deep learning systems for TED diagnosis, particularly in the automated analysis of ocular inflammation, eyelid retraction, and ocular motility disorders, achieving diagnostic accuracy rates of 95.1% for upper eyelid retraction and 87.0% for lower eyelid retraction (27). The application of artificial intelligence technology in TED diagnosis and treatment has promising prospects. AI algorithms can automatically measure palpebral fissure parameters, grade the severity of upper eyelid retraction, analyze imaging data to predict disease progression and treatment response, and assist in surgical planning and prognosis evaluation. These technologies will enhance diagnostic and therapeutic efficiency and precision.

## 5 Non-surgical treatment for upper eyelid abnormalities

Non-surgical treatment is primarily indicated for patients in the active phase, those who cannot tolerate or prefer not to undergo surgery, and as transitional therapy prior to surgical intervention. Patients with mild upper eyelid retraction without significant exposure keratitis may receive conservative management, including ocular surface protection measures such as artificial tears, eye ointments, and nocturnal coverage. For patients with moderate-to-severe upper eyelid abnormalities, local injection therapy (**Table 1**) and systemic pharmacotherapy (**Table 2**) have achieved significant advancements in recent years.

**Table 1:** Comparison of different local injection agents for TED upper eyelid abnormalities.

Agent	Mechanism	Efficacy (MRD1 reduction)	Duration	Common adverse effects	Level of evidence
BTX-A	Chemodenervation of LPS-SR	~1–2 mm	3–6 months	Ptosis, diplopia, rare globe injury	Moderate (multiple case series)
HA	Volumetric filler, mechanical barrier	~1–2 mm	Up to 78 months (variable)	Eyelid irregularity, overcorrection	Moderate (prospective trials)
TA	Anti-inflammatory, anti-fibrotic	~0.6-1.1 mm	6 months (may require repeat)	Elevated IOP, Cushingoid features	Moderate (controlled studies)
BTX-A + TA	Synergistic	Superior to monotherapy	Not well defined	Lower than TA alone	Low (small case series)
TA + Chinese acupuncture	Synergistic	~1–2 mm	Not well defined	Eyelid swelling	Low (small case series)
TA + herbal medicine	Synergistic	~2 mm	Not well defined	Diarrhea, eyelid swelling	Low (small case series)

**Table 2:** Comparison of different systemic pharmacotherapies for TED upper eyelid abnormalities.

Drug	Mechanism	Main efficacy	Common adverse effects	Level of evidence
Glucocorticoids	Broad anti-inflammatory, immunosuppressive	Improves eyelid edema and congestion	Weight gain, hypertension, hyperglycemia, osteoporosis, insomnia	High (first-line for active moderate-severe TED)
α-1 receptor antagonist (tamsulosin)	Blocks sympathetic tone on Müller’s muscle	Modest improvement in MRD1 (~1.04 mm) and palpebral fissure height	Dizziness, hypotension, rhinitis	Low (small prospective pilot study)
Teprotumumab (IGF-1R antagonist)	Blocks IGF-1R, inhibits TSHR cross-talk, and reduces orbital fibroblast activation	Significant reduction in eyelid retraction (MRD1), proptosis, and eyelid aperture	Ototoxicity (mostly transient), hyperglycemia (lower risk than steroids), fatigue, and muscle spasms	High (phase III RCT, real-world studies)
Sirolimus (mTOR inhibitor)	Antiproliferative, antifibrotic, immunosuppressive	May reduce proptosis and CAS	Mouth ulcers, hyperlipidemia, cytopenias, hypertension	Very low (small comparative study vs. steroids)
Linsitinib (IGF-1R/IR inhibitor)	Oral bispecific inhibitor of IGF-1R and insulin receptor	Positive in mouse model; no human data on eyelid retraction yet	Hypoglycemia, fatigue	Preclinical only (murine model)
Atorvastatin (statin)	Anti-inflammatory, immunomodulatory	Adjunctive benefit in TED (reduces progression)	Myalgia, elevated transaminases, and diabetes risk	Low
Selenium (antioxidant)	Reduces oxidative stress and modulates the immune response	Improves quality of life and slows progression in mild TED	GI upset, garlic breath, rare selenosis	Moderate (positive RCT for mild TED)

### 5.1 Local drug injection

#### 5.1.1 Botulinum toxin type A

Botulinum toxin type A (BTX-A) induces reversible chemical denervation by inhibiting acetylcholine release at the neuromuscular junction, thereby causing muscle relaxation and paralysis. BTX-A injections are typically administered via routes: transconjunctival and transdermal. Since Scott first reported the use of BTX-A via the upper eyelid skin for treating TED-associated upper eyelid retraction in 1973, this technique has been widely adopted in clinical practice (28, 29). Subsequently, BTX-A injection has been employed as a non-surgical treatment for upper eyelid abnormalities in patients with TED to weaken the LPS-SR complex and induce myogenic ptosis. Ozturk Karabulut et al. (30) reported single subcutaneous BTX-A injections in 60 patients with hypothyroidism-related ptosis, achieving acceptable cosmetic outcomes in the 58 of 71 eyes (81.7%), particularly in alleviating eyelid retraction. Biglan (31) (two patients) and Ozkan et al. (32) (four patients) reported six cases of hypothyroidism-induced upper eyelid retraction treated via transdermal BTX-A injection targeting the LPS-SR complex, yielding satisfactory results. Although current studies demonstrate an efficacy rate exceeding 90% for transdermal BTX-A injection in treating TED-associated upper eyelid abnormalities, the actual efficacy may be lower than reported because of the lack of controlled experimental groups in most studies and the fact that some TED patients exhibit spontaneous resolution of eyelid abnormalities. It is unclear whether the reduction in eyelid position is entirely attributable to BTX-A. Furthermore, no study has definitively established a dose–response relationship between BTX-A injection dose and the degree of upper eyelid retraction (33). Previously, Uddin and Davies (34) reported that subconjunctival BTX-A injection yielded superior efficacy compared with previously reported methods, likely due to its more precise and reproducible targeting of the levator palpebrae superioris and Müller’s muscle, rather than the relatively blind transdermal injection into the levator region employed in earlier studies. Additionally, Uddin and Davies highlighted another advantage of the subconjunctival approach: a reduced risk of post-injection motor dysfunction compared with transdermal injection. Domestic ophthalmologists also consider the subconjunctival route to be safer and more effective injection method (35, 36).

The most common complication of BTX-A injection is ptosis; additional potential complications include vertical strabismus, undercorrection or overcorrection, penetrating globe injury, optic neuropathy, and pupillary rigidity (37). Leung et al. (38) reported the case of a 67-year-old TAO patient who developed a penetrating globe injury following BTX-A injection, with immediate pupillary dilation of 8 mm in the injected eye and subsequent glaucoma symptoms. Optic neuropathy is a rare but severe complication of BTX-A, with only isolated case reports to date. However, patients typically return to baseline status after oral corticosteroid therapy. Both transdermal and transconjunctival injections require repeated administration to maintain efficacy and carry certain toxicity risks, leading to challenges in patient compliance.

#### 5.1.2 Hyaluronic acid filler

Hyaluronic acid (HA) fillers have been increasingly used in recent years to correct upper eyelid retraction. Their mechanism of action involves creating a physical barrier between the levator palpebrae superioris aponeurosis and tarsal plate through volumetric filling, mechanically limiting the upward lifting force of the eyelid (39). HA fillers possess numerous advantageous properties that make them widely preferred as injectable fillers. Their extremely low immunogenicity and relative ease of application have established them as the most commonly used injectable filler today (40). Additionally, some studies have demonstrated that HA is effective in improving eyelid retraction caused by temporal eyelid depression. The HA is distributed around the levator palpebrae superioris muscle, primarily located in the anterior aponeurotic space. At this site, HA adapts its morphology in response to eyelid movements and may reduce friction during interactions with adjacent tissues. This bursa-like effect is likely a key mechanism underlying the enhanced eyelid mobility and improved eyelid positioning following HA injection (41). Research by Young et al. confirmed that the therapeutic effects of HA were most pronounced immediately after injection. A prospective trial by Kohn et al. (42) showed an average reduction of 1 mm in medial rectus muscle displacement (MRD1) 1 month after HA injection, with the effect persisting for an average of 15 months. Compared with Kohn’s study, Young et al. reported a greater average reduction in MRD1 following a single HA injection of approximately 2 mm. The study also found that the anti-ptosis effect of the HA injection persisted for several months, up to 78 months. The primary advantages of HA injection include simple operation, immediate visible effects, absence of neurotoxicity, reversibility (dissolvability with hyaluronidase), and low immunogenicity. Compared with BTX-A, HA does not cause complications such as ptosis or diplopia. However, precise dosage and injection site control are required because excessive administration may lead to eyelid irregularities (43). In addition, HA requires repeated injections to maintain its therapeutic efficacy.

#### 5.1.3 Triamcinolone acetonide

Triamcinolone acetonide (TA) is a long-acting corticosteroid that improves eyelid retraction and alleviates eyelid edema and congestion through its anti-inflammatory and antifibrotic effects. Recent studies have demonstrated that local TA injection effectively reduces the degree of upper eyelid retraction in patients with thyroid eye disease (TED) during both active and stable phases. The primary injection sites were in the subconjunctival and periorbital regions. The mechanisms by which TA injection addresses TED-related upper eyelid abnormalities include its anti-inflammatory effects on the levator palpebrae superioris muscle and Müller’s muscle, steroid-induced myopathy, degenerative changes in the levator palpebrae superioris muscle, separation of the levator palpebrae superioris aponeurosis from the tarsal plate, or steroid-induced Müller’s muscle atrophy (44). Li et al. (45) and Young et al. (46) found that TA injection significantly mitigated inflammatory signs such as eyelid retraction edema or congestion. Lee et al. (47) reported an average reduction of 0.6–1.1 mm in medial rectus diameter (MRD) over a mean follow-up period of 6 months. Some patients required multiple injections, suggesting a potential need for repeated administration to achieve optimal control. Studies indicate that TA injection may offer greater advantages for patients in the active phase of TED (48). Lee et al. also observed more pronounced improvements in the active group (86.3%) compared than in the inactive group (25%). Li Dongmei et al. found that combined periorbital and superior fornix TA injection yielded more significant improvements in eyelid retraction and eyelid congestion/edema than periorbital TA injection alone.

Local injection of TA effectively alleviated upper eyelid abnormalities in patients with TED. For patients in the active phase, TA improves upper eyelid abnormalities by suppressing local inflammation. For those in the non-active phase, TA exerts its therapeutic effect by releasing fibrous adhesions. The most common adverse effect is elevated intraocular pressure, although all reported cases indicate that this increase is controllable and transient. Other complications included hemorrhage, hematoma, ptosis, menstrual cycle disorders, and crescent face formation, with the latter two resolving within months post-injection. Owing to its hormonal properties and the requirement for multiple injections, many patients develop significant aversion to the side effects of corticosteroids as medical knowledge has advanced.

#### 5.1.4 Other local injections

Given the respective advantages and disadvantages of TA and BTX-A when administered individually, Huang et al. (49) in recent years adopted a combined approach of subconjunctival BTX-A injection combined with periorbital TA injection for treating upper eyelid abnormalities caused by TED, achieving favorable outcomes with significantly lower complication rates than TA alone. This combined injection method enhances therapeutic efficacy and reduces complications, representing an innovative advancement over previous approaches. Xiong et al. (50) investigated the therapeutic effect of conjunctival injection of medical chitin on TED-related upper eyelid abnormalities and found that medical chitin effectively promoted recovery. As a natural product, medical chitin is metabolized into carbon dioxide and water in the body, exhibiting excellent biodegradability. Clinically, it is primarily used to prevent and treat intraoperative and postoperative adhesions by including reducing inflammatory cytokine production and inhibiting fibroblast proliferation (51). This represents a viable treatment option for patients intolerant to hormonal or botulinum toxin therapies. With advancements in integrated traditional Chinese and Western medicine research, Zhu et al. (52) demonstrated superior outcomes in treating TED-associated upper eyelid retraction using conjunctival triamcinolone acetonide combined with traditional Chinese acupuncture compared with either therapy alone. Similarly, Yang et al. (53) reported significant efficacy of periorbital triamcinolone acetonide combined with herbal medicine for TED-related upper eyelid retraction. These findings indicate that integrated Chinese-Western medical therapy holds promise as a key direction for future research.

### 5.2 Systemic pharmacotherapy

#### 5.2.1 Glucocorticoids

Intravenous glucocorticoid pulse therapy is currently the first-line treatment for moderate-to-severe active TED (53). Numerous studies have indicated that systemic glucocorticoid therapy demonstrates therapeutic efficacy for upper eyelid abnormalities, including edema, congestion, and eyelid retraction. The European Group on Graves’ Orbitopathy (EUGOGO) guidelines further recommend a combination of mycophenolate mofetil and corticosteroid therapy to enhance therapeutic outcomes. High-dose methylprednisolone pulse therapy effectively alleviated upper eyelid inflammation and edema, potentially indirectly improving eyelid positioning abnormalities caused by edema and inflammation. However, some researchers argue that glucocorticoid pulse therapy has limited efficacy in treating TED-induced eyelid retraction and has significant systemic side effects (such as weight gain, hypertension, elevated blood glucose, and osteoporosis). The effectiveness of glucocorticoid therapy in improving eyelid retraction varies between individuals. However, glucocorticoid pulse therapy remains the first-line treatment for upper eyelid abnormalities in patients with moderate-to-severe thyroid eye disease (54).

#### 5.2.2 IGF-1R antagonist

In recent years, the newly emerged biologic agent Teprotumumab, as an IGF-1R antagonist, has garnered widespread attention due to its ability to specifically block the IGF-1 receptor. In a double-blind, multicenter randomized trial published in 2017 (55), 88 patients with moderate-to-severe active Graves’ ophthalmopathy were randomly assigned to receive either placebo or Teprotumumab treatment. At 24 weeks, 29 out of 42 patients (69%) in the Teprotumumab group achieved remission, compared with only 9 out of 45 patients (20%) in the placebo group. The most significant therapeutic effects were observed for reduced proptosis and decreased palpebral fissure height. A subsequent Phase III randomized clinical trial enrolled 41 patients treated with Teprotumumab and 42 patients in the placebo control group, demonstrating an 83% remission rate in the Teprotumumab group versus only 10% in the placebo group. Moreover, a recent placebo-controlled, double-blind clinical trial in Japan showed that after eight injections, the majority of patients treated with Teprotumumab experienced reductions in proptosis, orbital fat, and extraocular muscle cross-sectional area on MRI, as well as in the degree of inflammation/edema (56). Due to its remarkable therapeutic efficacy, the American Academy of Ophthalmology (ATA) and the European Academy of Ophthalmology (ETA) issued a consensus in 2022 recommending Teprotumumab as the first-line treatment for TED-related proptosis and diplopia (57). Its mechanism likely involves inhibiting the cross-talk between IGF-1R and TSHR on ocular fibroblasts (OFs), thereby fundamentally reducing inflammation and glycosaminoglycan production. IGF-1R antagonists have emerged as pivotal agents transforming the treatment landscape for TED, offering a novel therapeutic option for patients who are ineligible for or respond poorly to glucocorticoids. However, clinical summaries indicate potential adverse effects such as ototoxicity, hyperglycemia, and inflammatory bowel disease (58). However, a recent large-scale retrospective study suggested that the ototoxicity caused by Teprotumumab is mostly transient and does not require specific treatment. Moreover, the risk of hyperglycemia induced by Teprotumumab is lower than that associated with conventional corticosteroid therapy (59). While Teprotumumab has demonstrated efficacy in addressing TED-induced upper eyelid abnormalities, ongoing research continues to evaluate its clinical effectiveness in such patients owing to its high cost and limited long-term efficacy data.

#### 5.2.3 α-1 receptor antagonist

Based on the theory that Müller’s muscle is innervated by the sympathetic nervous system, α-1 adrenergic receptor antagonists theoretically block the effects of sympathetic excitation on Müller’s muscle, thereby alleviating eyelid abnormalities such as upper eyelid retraction. Arnon (60) and colleagues conducted a prospective preliminary study involving 11 patients with TED-related eyelid retraction who were treated with the oral α-1 receptor antagonist tamsulosin. The results showed objective improvement in eyelid position in five patients, with an average reduction of 1.04 mm in mean MRD and 1.46 mm in mean palpebral fissure height. Tamsulosin may serve as a temporary alternative treatment for patients who cannot tolerate surgical interventions. However, owing to the small sample size and non-controlled design of the study, the findings should be interpreted with caution. The efficacy of α-1 antagonists in treating TED-related upper eyelid abnormalities, particularly eyelid retraction, requires validation in large-scale controlled studies.

#### 5.2.4 Other systemic agents

Rituximab is a human–mouse chimeric monoclonal antibody that binds to CD20+ B cells and induces their apoptosis (61). In early studies, some researchers suggested that rituximab might be effective for moderate-to-severe active Graves’ ophthalmopathy (62); however, a subsequent randomized clinical trial demonstrated no significant benefit of rituximab compared with placebo (63). Sirolimus is an antiproliferative, antifibrotic, and immunosuppressive agent used to prevent organ rejection. A comparative study between sirolimus and methylprednisolone treatment showed that the sirolimus group exhibited a higher proportion of patients with reduced proptosis and improved CAS scores compared with the methylprednisolone group (64). However, due to limitations in the study design and sample size, further randomized clinical trials are warranted. Linsitinib is an oral bispecific small-molecule drug that inhibits IGF-1R. In 2023, Gulbins et al. reported its positive efficacy in a murine model of Graves’ ophthalmopathy (65), and its therapeutic potential in patients with TED-associated upper eyelid abnormalities warrants further investigation. Atorvastatin (66) and selenium (67) may serve as adjunctive therapies for TED-associated upper eyelid abnormalities because of their beneficial effects in patients with TED.

## 6 Surgical treatment of upper eyelid abnormalities

For patients with upper eyelid abnormalities in TED who respond poorly to conservative treatment, surgical intervention is the only option (**Table 3**). Surgery for TED-related upper eyelid abnormalities, particularly eyelid retraction, is typically performed when the condition is stable (i.e., during the quiescent phase), including when thyroid function is normal and stable, without significant proptosis or strabismus (or when proptosis and strabismus have been corrected), and when the degree of eyelid retraction is stable. Some researchers advocate performing orbital decompression and eyelid retraction correction surgeries concurrently (68). As surgeries on the superior and inferior rectus muscles affect the positions of the upper and lower eyelid margins, respectively, eyelid retraction correction should be performed after extraocular muscle surgery. Studies have reported that 70% of patients with upper eyelid retraction experience spontaneous improvement within 12 months, 75% within 24 months, 33.3% return to normal within 12 months, and 50% within 24 months (69). Therefore, surgical treatment should be considered for at least 18 months after the onset of eyelid retraction, preferably after 24 months (70).

**Table 3:** Indications and level of evidence for different surgical procedures for TED upper eyelid retraction.

Procedure	Indications	Advantages	Disadvantages	Level of evidence
Müller muscle and levator palpebrae superioris muscle surgery	Eyelid retraction of all degrees	Simple, short recovery, adjustable, effective	Unpredictable outcome; possible under- or overcorrection	Moderate (case series)
Spacer graft (implant)	Severe retraction, failed prior surgery	Effective for large corrections	Graft complications (thickening, cyst)	Low to moderate
Blepharotomy	Eyelid retraction and eyelid swelling of all degrees	Highly predictable, adjustable postop	dry eye syndrome	Moderate (increasing evidence)
Orbital decompression + eyelid surgery	Proptosis with eyelid retraction	Addresses both issues in one stage	Higher complexity, risk of diplopia	Moderate (comparative studies)

### 6.1 Müller muscle and levator palpebrae superioris muscle surgery

The dissection or surgical technique involving the Müller muscle and levator palpebrae superioris was first proposed by Henderson (71) in 1965 and has since been refined by numerous researchers to achieve superior outcomes. These techniques include Müller muscle resection, levator palpebrae superioris recession, combined Müller muscle resection with levator recession, levator palpebrae superioris and Müller muscle lengthening, and modified central aponeurotomy of the levator palpebrae superioris with Müller muscle transection. To date, however, no single technique has been proven to be more effective or stable than others. These procedures can be performed either through the skin or conjunctiva, typically requiring only local anesthesia, and are characterized by simplicity and short operative duration (72). Pinas (73) and colleagues presented a case series report on Müller muscle and levator palpebrae superioris lengthening surgery, noting that although patients exhibited improved symptoms postoperatively, the outcomes remained highly unpredictable. Moreover, regardless of surgical experience or prior interventions, approximately 20% of patients remained dissatisfied with the surgical results.

### 6.2 Implant surgery

Implant surgery may serve as a viable option for patients with severe TED blepharoptosis or those who have achieved suboptimal outcomes with other surgical approaches, implant surgery may serve as a viable option. Callahan pioneered the use of septa to treat TED blepharoptosis (74); he advocated retracting the levator palpebrae superioris muscle by 10 mm and employing the tibialis anterior muscle or a collagen membrane as the septum. Subsequently, his team further proposed that scleral grafts represent the optimal choice for implant surgery (75). Since then, various septal grafts, including stratified auricular cartilage, stored sclera, and broad fascia—have been widely utilized. However, graft-related complications such as eyelid thickening and cysts may affect ocular appearance (76). Sun et al. (77) employed bovine acellular dermal matrix as an improved septal graft for levator palpebrae superioris lengthening, achieving simultaneous improvement in severe TED-related blepharoptosis symptoms and signs with fewer complications.

### 6.3 Blepharotomy

Blepharotomy has been recognized as the most effective surgical technique in recent years, following refinements by researchers (77). Its advantages include the use of local anesthesia, relative simplicity, short operative time, absence of implant or suture traction requirements, applicability to all degrees of upper eyelid retraction, excellent predictability of upper eyelid height and contour, no need for intraoperative over-correction, and stable palpebral fissure height. The same technique can be employed for reconstructive surgery. Within 2 weeks postoperatively, patients can adjust upper eyelid height and shape through traction and massage (78). Some patients with TED-associated upper eyelid abnormalities continue to exhibit symptoms such as eyelid skin swelling after treatment, which may be attributed to increased orbital fat volume, enlargement of suborbicular muscle fat, and expansion of the suborbicular fat pad, leading to brow elevation (69). TED eyelid reconstruction surgery is typically performed during the non-acute phase of TED, or following other surgical procedures. Some researchers advocate performing orbital decompression surgery concurrently with cosmetic eyelid surgery (79). Excessive tissue, such as partial suborbicular or suborbicular muscle fat, can be appropriately removed with a double eyelid incision to ensure aesthetic outcomes.

### 6.4 Orbital decompression surgery

Orbital decompression surgery can improve upper eyelid abnormalities by alleviating proptosis symptoms (80). For patients with TED presenting with both proptosis and upper eyelid retraction, some researchers have begun to consider the possibility of simultaneously performing orbital decompression and upper eyelid retraction correction surgeries simultaneously. Simon et al. (81) conducted the first comparative study comparing concurrent orbital decompression and ptosis correction surgery with staged surgery and found similar efficacy outcomes in both groups. Quaranta-Leoni et al. (82) performed another retrospective study comparing combined orbital decompression, eyelid, and strabismus surgery with staged surgery and demonstrated no significant differences in MRD1 or postoperative diplopia between the groups. These studies indicate that combining upper eyelid correction surgery with orbital decompression yields favorable prognostic outcomes in patients with severe TED-associated upper eyelid abnormalities, warranting further investigation.

## 7 Conclusions

Research on the treatment of upper eyelid abnormalities in thyroid eye disease has evolved over nearly a century, establishing a comprehensive therapeutic framework that includes conservative management, local injection, systemic pharmacotherapy, and surgical correction. In the field of pharmacotherapy, the introduction of novel agents such as IGF-1R antagonists, lisitinib, and sirolimus has expanded the therapeutic scope beyond glucocorticoid therapy, offering patients with new treatment options with significant efficacy. Regarding surgical interventions, techniques such as implant placement, blepharotomy, and orbital decompression combined with eyelid correction have been continuously refined for TED epicanthal retrusion in the quiescent phase or when conservative treatments are ineffective. The selection of optimal surgical timing has also progressively improved, making these procedures viable alternatives when non-surgical therapies yield suboptimal outcomes.

Despite significant progress in the diagnostic and therapeutic research on TED epiphthalmos, numerous challenges remain unresolved. These include the lack of comparative studies among different treatment modalities such as BTX-A and TA, the absence of large-scale, multicenter, controlled evidence-based studies on medical chitosan injections and integrated traditional Chinese and Western medicine therapies, and the insufficient clinical application data supporting emerging targeted biologics. The optimal timing of treatment and combination strategies remain controversial—issues such as the delineation between active and quiescent phases for treatment selection, and the sequential or combined use of biologics with local injections or surgical interventions—all require further standardization through extensive clinical trials. In the future, in-depth elucidation of signaling pathways represented by IGF-1R and TSHR will lead to the development of more efficient and low-adverse-effect targeted biologics. Artificial intelligence technologies will be further integrated into image quantification, surgical planning, and prognosis prediction. Multidisciplinary team (MDT) collaboration models across ophthalmology, endocrinology, radiology, and radiation oncology have been widely adopted. Consequently, the diagnosis and treatment of TED epiphthalmos will become more precise, targeted, intelligent, and holistic, enabling clinicians to deliver optimal treatment timing and protocols to diverse patient populations.
